# Indications for enzymatic denitrification to N_2_O at low pH in an ammonia-oxidizing archaeon

**DOI:** 10.1038/s41396-019-0460-6

**Published:** 2019-06-21

**Authors:** Man-Young Jung, Joo-Han Gwak, Lena Rohe, Anette Giesemann, Jong-Geol Kim, Reinhard Well, Eugene L. Madsen, Craig W. Herbold, Michael Wagner, Sung-Keun Rhee

**Affiliations:** 1 Department of Microbiology, Chungbuk National University, 1 Chungdae-ro, Seowon-Gu, Cheongju, 28644 South Korea; 2 University of Vienna, Centre for Microbiology and Environmental Systems Science, Division of Microbial Ecology, Althanstrasse 14, A-1090 Vienna, Austria; 3 Helmholtz Centre for Environmental Research—UFZ, Department of Soil System Sciences, Theodor-Lieser-Strasse 4, D-06120 Halle (Saale), Germany; 4 Thünen Institute of Climate-Smart Agriculture, Bundesallee 50, D-38116 Braunschweig, Germany; 5 Department of Microbiology, Cornell University, Ithaca, NY 14853-8101 USA; 6 The Comammox Research Platform, University of Vienna, Althanstrasse 14, A-1090, Vienna, Austria; 7 Department of Biotechnology, Chemistry and Bioscience, Aalborg University, Fredrik Bajers Vej 7H, 9220 Aalborg, Denmark

**Keywords:** Microbial ecology, Microbial ecology

## Abstract

Nitrous oxide (N_2_O) is a key climate change gas and nitrifying microbes living in terrestrial ecosystems contribute significantly to its formation. Many soils are acidic and global change will cause acidification of aquatic and terrestrial ecosystems, but the effect of decreasing pH on N_2_O formation by nitrifiers is poorly understood. Here, we used isotope-ratio mass spectrometry to investigate the effect of acidification on production of N_2_O by pure cultures of two ammonia-oxidizing archaea (AOA; *Nitrosocosmicus oleophilus* and *Nitrosotenuis chungbukensis*) and an ammonia-oxidizing bacterium (AOB; *Nitrosomonas europaea*). For all three strains acidification led to increased emission of N_2_O. However, changes of ^15^N site preference (SP) values within the N_2_O molecule (as indicators of pathways for N_2_O formation), caused by decreasing pH, were highly different between the tested AOA and AOB. While acidification decreased the SP value in the AOB strain, SP values increased to a maximum value of 29‰ in *N. oleophilus*. In addition, ^15^N-nitrite tracer experiments showed that acidification boosted nitrite transformation into N_2_O in all strains, but the incorporation rate was different for each ammonia oxidizer. Unexpectedly, for *N. oleophilus* more than 50% of the N_2_O produced at pH 5.5 had both nitrogen atoms from nitrite and we demonstrated that under these conditions expression of a putative cytochrome P450 NO reductase is strongly upregulated. Collectively, our results indicate that *N. oleophilus* might be able to enzymatically denitrify nitrite to N_2_O at low pH.

N_2_O is an important ozone-depleting substance with a high global warming potential [1–3]. The processes of biological N_2_O production include partial dissimilatory nitrate (NO_3_^−^) or nitrite (NO_2_^−^) reduction (denitrification), nitrifier denitrification [2], ammonia [hydroxylamine (NH_2_OH)] oxidation, and NOx detoxification (also known as the “nitrosative stress” pathway) [1]. AOB are recognized as a major source of N_2_O production from terrestrial environments [4] and AOA are also considered to be important contributors to N_2_O production in various environments, based on their high abundance in many ecosystems and the documented formation of N_2_O during AOA-mediated ammonia oxidation [5–8]. Despite the presence of a nitrite reductase gene (*nirK*) in almost all AOA genomes, canonical nitric oxide reductase (NOR) genes have not been detected in these organisms. Instead, isotope labeling experiments suggested hybrid N_2_O formation in the AOA *Nitrososphaera viennensis* from nitrite and an intermediate of ammonia oxidation, and was attributed to either an enzymatically catalyzed (codenitrification) or abiotic N-nitrosation reaction [8]. Under low-oxygen conditions, abiotic formation of N_2_O from hydroxylamine or NO was observed in experiments with killed *N. viennensis* biomass in AOA media [9]. Furthermore, it was recently demonstrated that N_2_O is formed abiotically under aerobic conditions from hydroxylamine and nitrite produced by aerobic ammonia-oxidizing microbes and that this hybrid pathway can account for a large proportion of the aerobically produced N_2_O [10]. Interestingly, we observed in a previous study for selected AOA strains variable N_2_O isotopomer SP (enrichment of ^15^N at the alpha, or beta site of N_2_O) values (ca. 20–30‰) and a very variable contribution of both N atoms in N_2_O from nitrite (8.4–53% of the N_2_O produced by the AOA strains had both N from nitrite) at high initial nitrite concentrations. These results are consistent with the hypothesis that nitrifier denitrification might contribute to N_2_O production by some AOA in the presence of excess nitrite [6].

Terrestrial and ocean environments experience acidification due to natural or human activities [11, 12] and a large part (ca. 30%) of the world’s natural and arable soils are acidic (pH < 5.5) [13]. However, most of the studies of N_2_O production by AOA and AOB have been performed in circumneutral pH ranges only, although various biological and chemical reactions involved in N_2_O production are pH-dependent [11]. Thus, responses of N_2_O production by AOA and AOB to acidification need to be better understood to estimate the present and future contribution of soil N_2_O production to the global N_2_O budget. The present study was designed to reveal the impact of acidification upon N_2_O production by two selected AOA strains and by a model strain of AOB. We analyzed N_2_O production and SP of the N_2_O molecule under varying pH conditions. In addition, pH-dependent changes of the source of nitrogen of N_2_O were investigated using ^15^N-nitrite tracer experiments in order to obtain insights into potential differences of the N_2_O production pathways between the analyzed strains.

In order to assess the effect of acidification on N_2_O production, axenic AOA (*Nitrosotenuis chungbukensis* MY2 of thaumarchaeal group I.1a; *Nitrosocosmicus oleophilus* MY3 of thaumarchaeal group I.1b) and AOB (*Nitrosomonas europaea* ATCC 19718) strains were incubated in growth media spanning a pH range of 5.5–8.5 (at intervals of 0.5 pH units). More details on cultivation methods, media, and incubation conditions are described in the Supplementary Materials and Methods. The lowest pH used in these assays was selected after screening the three cultures for retention of ∼20% of the growth rate found at optimal pH. Thus, pHs of 5.5, 6.0, and 6.5 were used as most acidic incubation conditions for *N. oleophilus* MY3, *N. chungbukensis* MY2, and for *N. europaea* ATCC 19718, respectively. For all tested ammonia oxidizers the N_2_O yield (N_2_O-N/oxidized NH_4_^+^-N) increased as pH decreased (Supplementary Fig. S1). The N_2_O yield of *N. chungbukensis* MY2 at low pH was much higher than that of *N. oleophilus* MY3. The increased N_2_O production at acidic conditions may be caused by increased production of enzymes involved in N_2_O production [14], an acidic pH optimum of N_2_O-producing enzymes [15, 16], or acceleration of abiotic hybrid N_2_O formation [8, 17]. In coculture experiments of the AOA *N. oleophilus* MY3 with the nitrite-oxidizing bacterium (NOB), *Nitrobacter winogradskyi* Nb-255 nitrite accumulation was not observed during nitrification at pH 7.5 and pH 5.5 (Supplementary Fig. S2). This *Nitrobacter* strain has no known enzymatic repertoire to produce or oxidize N_2_O, but encodes a reversible nitrite oxidoreductase that is able to catalyze the oxidation of nitrite to nitrate [18]. Interestingly, for both AOA and AOB at the lowest pH tested, the presence of the NOB in the cultures caused a significant decrease of N_2_O yields (28% and 48%, respectively) (*P* < 0.001), suggesting that the accumulation of nitrite in the experiments without addition of NOB contributed to the increased N_2_O production under these conditions (Supplementary Fig. S1). Thus, it is tempting to speculate that the higher N_2_O production at lower pH may possibly be connected to an upregulation of nitrite detoxification due to increased formation of the reactive compound nitrous acid (HNO_2_) from nitrite (NO_2_^−^, pKa = 3.39) at lower pH.

Many isotopic studies have documented a positive SP value (around 30‰) that is the characteristic for N_2_O produced by ammonia oxidizers via the formation of NH_2_OH [19], with similar values reported for fungal denitrification [20] or chemical formation of N_2_O from hydroxylamine [21]. In contrast, SP values for N_2_O produced by bacterial heterotrophic denitrification or nitrifier denitrification are both near or below zero [19]. The SP values of N_2_O produced by the strains in the present study (*N. oleophilus* MY3, *N. chungbukensis* MY2, and *N. europaea* ATCC 19718) at pH 7.5 were 26‰, 29‰, and 28‰, respectively, resembling the signatures of N_2_O produced mostly via NH_2_OH formation (Fig. 1a). The SP values of N_2_O produced by the AOB strain ATCC 19718 decreased dramatically from ~30‰ at pH 8.5 to ~0.5‰ (*P* < 0.001) at pH 6.5 (Fig. 1a). This shift is consistent with prior observations that increased nitrifier denitrification activity of AOB at decreasing pH [14] may be associated with removal of HNO_2,_ the toxic form of NO_2_^-^. In contrast, for *N. chungbukensis* MY2 only a slight decrease of SP values of N_2_O (from 29 to 27‰; *P* = 0.05) was observed when the pH dropped from pH 7.5 to 6.0 (Fig. 1a). Intriguingly, for *N. oleophilus* MY3 the SP values of N_2_O even increased from 26 to 29‰ (*P* < 0.05), when the pH decreased from 7.5 to 5.5 suggesting no involvement of canonical nitrifier denitrification. Meanwhile, continuous nitrite removal from the *N. oleophilus* MY3 culture by cocultivation with a NOB had no significant effect on the SP values of N_2_O at pH 5.5 (Fig. 1a) indicating independence of N_2_O production mechanisms on the external nitrite concentration at low pH. Our observations of opposing and/or variable trends in SP values of N_2_O in the different strains as a function of pH indicates that, although N_2_O yields increased under acidic conditions in the tested AOA and AOB cultures (see Supplementary Fig. S1), the underlying mechanisms responding to acidification likely to differ.

**Fig. 1:**
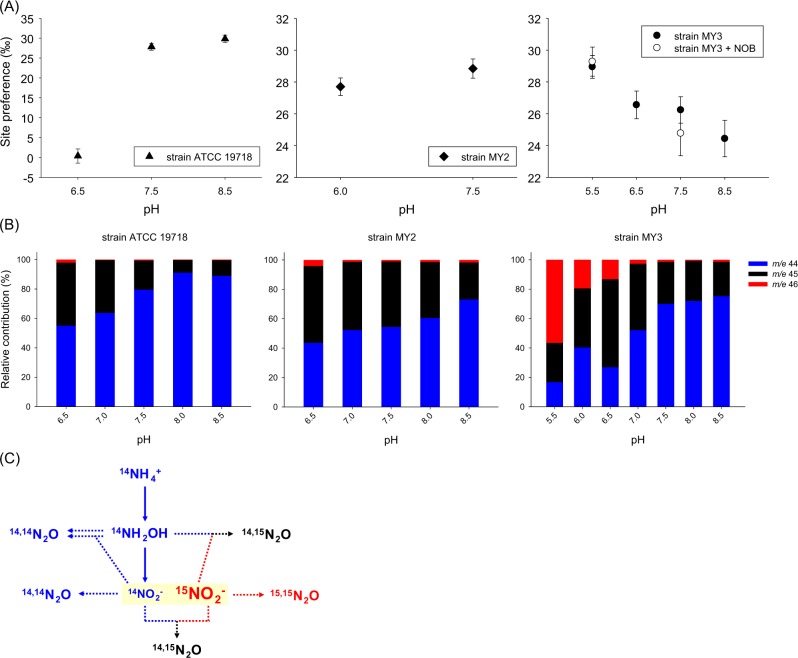
**a** ^15^N site preference (SP) values of N_2_O at various pH conditions for *N. europaea* ATCC 19718, *N. chungbukensis* MY2, and *N. oleophilus* MY3. The SP values of N_2_O were measured after ammonia oxidation was completed in the incubation experiments. *Nitrobacter winogradskyi* Nb-255 was the cocultured NOB. The error bars are based on replicate experiments to show the standard deviation and the raw data used in this plot are presented in Supplementary Table S3. **b** The panels depict the composition of labeled N_2_O produced during the tracer experiment by *N. europaea* ATCC 19718, *N. chungbukensis* MY2, and *N. oleophilus* MY3. The ammonia oxidizers were incubated in the presence of 0.2 mM unlabeled NH_4_^+^ and 0.2 mM ^15^N-labeled nitrite at different pH conditions. The values for the different masses of N_2_O at each pH condition for each strain are presented as mean values of triplicate experiments (standard deviations of all values were <5%). **c** Proposed (bio)chemical processes showing the pathways leading to N_2_O production in the tracing experiment. Two different nitrogen sources (from unlabeled-ammonia or ^15^N-labeled nitrite) permitted three possible mass combinations in the produced N_2_O. It should be noted that hydroxylamine is converted to NO by the hydroxylamine dehydrogenase in AOB and then further oxidized to nitrite by an unknown enzyme ([23]; not shown). NO has also been suggested as an important intermediate in the energy metabolism of AOA, but its exact role is still under debate ([9, 23]; not shown). Unlabeled N_2_O (*m/e* 44) can be produced enzymatically by AOB by conversion of NH_2_OH by cytochrome P460 [29] or chemically in the presence of Fe^3+^ or Mn^4+^ [30]

The SP value of the N_2_O produced by the tested AOA does not exclude production of N_2_O by a chemical reaction of NO_2_^−^ with NH_2_OH during ammonia oxidation [10], as the SP of N_2_O abiotically produced from NH_2_OH and NO_2_^−^(or HNO_2_) at acidic conditions was reported to be ∼34‰ [21]. However, in our abiotic control experiments at pH 5.5 and 7.5, N_2_O production was much lower in the presence of 500 µM of NO_2_^−^with or without addition of 10 and 50 µM of NH_2_OH compared to corresponding experiments with active AOA and AOB cultures (see Supplementary Table S1 and 2). The NH_2_OH concentrations in the abiotic controls were selected based on the recently published data that showed maximum concentrations of extracellular NH_2_OH of <10 µM in AOA, AOB, and comammox strains during oxidation of 2 mM ammonia [10]. In this context it should be noted that previously higher N_2_O production in abiotic control incubations that we report here has been detected [9, 10]. This difference likely reflects differences in media composition (for example, we used a ~ 10 × lower trace metal concentration than Kozlowski et al. [9] and Liu et al. [10] for our biotic and abiotic experiments) and/or incubation conditions (e.g., our abiotic experiments were performed fully aerobic in contrast to Kozlowski et al. [9]) between studies.

The increase of the N_2_O yield by both tested AOA (and the increase of the SP value of N_2_O for *N. oleophilus* MY3) at lower pH cannot be explained by conventional nitrifier denitrification for HNO_2_ detoxification as this would be expected to strongly lower the SP value (as observed for *N. europaea*). To further investigate the underlying processes of N_2_O production, nitrogen incorporation into N_2_O produced by the three nitrifiers was traced by using ^15^N-labeled nitrite. With this setup, most of *m/e* 44 (^14,14^N_2_O) and all of *m/e* 46 (^15,15^N_2_O) is produced by conversion of unlabeled ammonia and ^15^N-labeled nitrite, respectively (see Fig. 1b, c). Interestingly, the relative contribution of labeled nitrite to N_2_O production (*m/e* 45 – one labeled N atom + *m/e* 46) was increased by acidification in all strains (Fig. 1b).

For *N. europaea* ATCC 19718, the increased N_2_O yield and decreased SP at low pH suggests production of N_2_O via nitrifier denitrification by the combined action of its nitrite reductase and nitric oxide reductase enzymes. Dissolved O_2_ concentrations in our cultures of AOA and AOB were >95% saturation and those enzymes are also known to be expressed under aerobic conditions [14, 22]. Unexpectedly however, in the ^15^N-nitrite labeling experiment double-labeled N_2_O (*m/e* 46) was not much increased at low pH and instead more hybrid-N_2_O formation (*m/e* 45) was observed (Fig. 1b). It is important to keep in mind that *N. europaea* ATCC 19718 produces much more NO than AOA at circumneutral pH [9], and the expression of nitrite reductase is further induced by low pH and high nitrite [14], which will lead to increased NO production from nitrite under these conditions. Furthermore, recent biochemical experiments demonstrated that the hydroxylamine dehydrogenase (HAO) of *N. europaea* produces NO and not nitrite during ammonia oxidation [23]. Consequently, in our labeling experiment at acidic conditions (unlabeled) ^14^NO formed from ammonia oxidation by the HAO activity will mix with (labeled) ^15^NO formed from nitrite via nitrite reductase. Reduction of this partially labeled NO by NorB (or NorSY [24], or cytochrome c554 [25]; with possibly different contributions of the different NO reductases with varying pH) will result in hybrid N_2_O formation (*m/e* 45). This hypothesis might also explain the results from recent stable isotope labeling experiments with a natural AOB community in a lake that also indicated increased hybrid N_2_O formation with decreasing pH [11].

For *N. chungbukensis* MY2, the nitrite-labeling experiment also suggested an increased contribution of hybrid-N_2_O formation (*m/e* 45) at pH 6.5 (Fig. 1b). Lowering the pH might have increased the N_2_O yield in *N. chungbukensis* MY2 by increasing chemical hybrid N_2_O formation from nitrite and hydroxylamine as previously described [10–12]. However, this is inconsistent with the data from our abiotic control experiments with nitrite and hydroxylamine that showed a much lower N_2_O production at pH 6.5 than measured in the corresponding biotic experiment. Thus, our data indicate that in *N. chungbukensis* MY2 N_2_O formation at low pH might be catalyzed by an unknown NO-reducing enzyme.

Surprisingly, for *N. oleophilus* MY3 up to 56.5% of the N_2_O produced in our labeling experiment had both N from nitrite (^15,15^N_2_O) implicating a substantial involvement of an unusual nitrifier denitrification process in N_2_O formation (Fig. 1b). In this context it is interesting to note that the SP values for N_2_O produced from fungal denitrification show values of up to 35‰ [19, 20], similar to those observed by us for *N. oleophilus* MY3 at low pH. The most characteristic feature of the fungal-denitrifying system is the involvement of cytochrome P450, as NOR (P450nor) [20, 26]. The proposed overall mechanisms for the reduction of NO by the enzyme P450nor is [2NO + NAD(P)H + H^+^ → N_2_O + H_2_O + NAD(P)^+^] and the enzyme accept two electrons directly from NAD(P)H, in contrast to other P450 enzymes (non-NOR type) where the electrons are donated one at a time via redox partners involving flavins and iron–sulfur centers [26]. The fungal denitrifying system seems to lack N_2_O reductase (NOS) and therefore evolves N_2_O as the final product [26]. Interestingly, putative cytochrome P450-encoding genes were detected by us in the genomes of *N. oleophilus* MY3 and some other thaumarchaeal group I.1b members, as well as in some nitrifying bacterial strains (AOB, NOB, and comammox) (Supplementary Fig. S3). The cytochrome P450 of *Nitrosocosmicus* spp., like the fungal P450nor clade (CYP55_NOR), is related to bacterial members of this enzyme superfamily. However, the fungal P450nor clade and the respective enzyme superfamily members of nitrifying bacteria, *Nitrososphaera* spp. and *Nitrosocosmicus* spp. are polyphyletic, and without biochemical data no direct proof of their specific enzymatic activity can be obtained. However, at pH 5.5 (compared to pH 7.5), we observed significantly increased transcription of two cytochrome P450-like genes (MY3_00641 and MY3_01637) of strain *N. oleophilus* MY3 compared to transcription of housekeeping genes (see Supplementary Table S4 for information on qPCR primers) such as those encoding the 16S rRNA, AmoA, and enzymes required for CO_2_ fixation (Fig. 2 and Supplementary Fig. S4). N_2_O yields are significantly increased due to nitrifier denitrification in AOB under low-oxygen conditions [8, 27, 28], which can be higher than those from nitrifier denitrification in *N. oleophilus* MY3 under aerobic conditions at acidic conditions (Supplementary Table S1). Altogether, this suggests that in *N. oleophilus* MY3 cytochrome P450 might be possibly involved in the production of N_2_O via nitrifier denitrification acting as NOR under aerobic conditions at a lower pH, a hypothesis that warrants further experimental investigation. If confirmed, cytochrome P450-catalyzed N_2_O production in AOA and possibly some other nitrifiers would significantly expand our perception of the metabolic repertoire of these important N-cycle microorganisms and their contribution to global change.

**Fig. 2:**
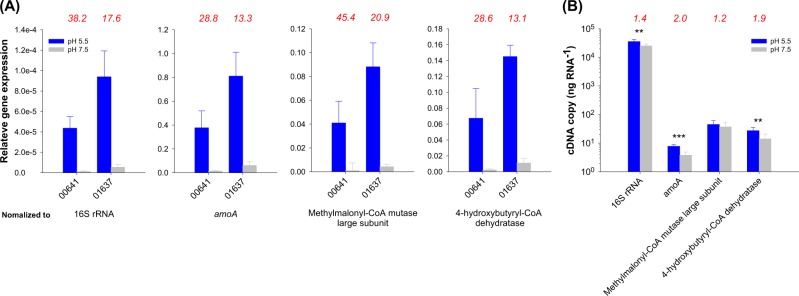
**a** *N. oleophilus* MY3 cytochrome P450 cDNA gene expression ratios at two different pH conditions (pH 5.5 and pH 7.5) normalized to other expressed genes. Average of two independent qPCR experiments performed on reverse-transcribed total RNA from cells grown at pH 5.5 and 7.5 and harvested at exponential stage are presented. Relative mRNA expression of two different copies of cytochrome P450 transcripts (locus: MY3_00641 and MY3_01637) to those of representative housekeeping genes [16S rRNA and mRNA of *amoA*, methylmalonyl-CoA mutase large subunit (MY_02370), and 4-hydroxybutyryl-CoA dehydratase (MY3_03315)], were calculated for obtaining normalized data. The ratio of relative expression at pH 5.5–7.5 is indicated above the graphs in red. Normalized expression of both P450 genes is significantly higher at low pH than at high pH in all panels (significance of differences of normalized expression level of cytochrome P450 gene between pH 5.5 and 7.5 were determined by a *t*-test (*P* < 0.05)). Error bars indicate standard deviation of duplicate experiments. The difference between the y-axes of the four panels should be noted. Expression at low pH between the two P450 genes was also found to be significantly different (*P* < 0.05). **b** Non-normalized RT-qPCR data cDNA for 16S rRNA, *amoA*, and methylmalonyl-CoA mutase large subunit (MY_02370) and 4-hydroxybutyryl-CoA dehydratase (MY3_03315) at pH 5.5–7.5. The cDNA transcripts of each gene were quantified per 1 ng of RNA. The ratio of relative expression at pH 5.5–7.5 is indicated above the graphs in red. Error bars indicate standard deviation from duplicate experiments. For each gene, significance of difference in measured cDNA copy number between pH 5.5 and 7.5 was determined by a *t*-test (*P < 0.5, **P < 0.1, and ***P < 0.05)

## Supplementary information

supplementary information

## References

[1] Stein LY (2011). Surveying N_2_O-producing pathways in bacteria. Methods Enzymol.

[2] Wrage N, Velthof GL, van Beusichem ML, Oenema O (2001). Role of nitrifier denitrification in the production of nitrous oxide. Soil Biol Biochem.

[3] Rahn T, Wahlen M (1997). Stable isotope enrichment in stratospheric nitrous oxide. Science.

[4] Gödde M, Conrad R (1999). Immediate and adaptational temperature effects on nitric oxide production and nitrous oxide release from nitrification and denitrification in two soils. Biol Fertil Soils.

[5] Löscher CR, Kock A, Könneke M, LaRoche J, Bange HW, Schmitz RA (2012). Production of oceanic nitrous oxide by ammonia-oxidizing archaea. Biogeosciences.

[6] Jung MY, Well R, Min D, Giesemann A, Park SJ, Kim JG (2014). Isotopic signatures of N_2_O produced by ammonia-oxidizing archaea from soils. ISME J.

[7] Santoro AE, Buchwald C, McIlvin MR, Casciotti KL (2011). Isotopic signature of N_2_O produced by marine ammonia-oxidizing archaea. Science.

[8] Stieglmeier M, Mooshammer M, Kitzler B, Wanek W, Zechmeister-Boltenstern S, Richter A (2014). Aerobic nitrous oxide production through N-nitrosating hybrid formation in ammonia-oxidizing archaea. ISME J.

[9] Kozlowski JA, Stieglmeier M, Schleper C, Klotz MG, Stein LY (2016). Pathways and key intermediates required for obligate aerobic ammonia-dependent chemolithotrophy in bacteria and *Thaumarchaeota*. ISME J.

[10] Liu S, Han P, Hink L, Prosser JI, Wagner M, Bruggemann N (2017). Abiotic conversion of extracellular NH_2_OH contributes to N_2_O emission during ammonia oxidation. Environ Sci Technol.

[11] Frame CH, Lau E, Nolan EJt, Goepfert TJ, Lehmann MF (2016). Acidification enhances hybrid N_2_O production associated with aquatic ammonia-oxidizing microorganisms. Front Microbiol.

[12] Heil J, Vereecken H, Brüggemann N (2016). A review of chemical reactions of nitrification intermediates and their role in nitrogen cycling and nitrogen trace gas formation in soil. Eur J of Soil Sci.

[13] von Uexküll HR, Mutert E. Global extent, development and economic impact of acid soils. In: Date RA, Grundon NJ, Rayment GE, Probert ME, (editors). Plant-Soil interactions at low pH: principles and management: proceedings of the third international symposium on plant-soil interactions at low pH. Brisbane, Queensland, Australia, 12–16 September 1993. Dordrecht: Springer Netherlands; 1995. p. 5–19.

[14] Beaumont HJ, Lens SI, Reijnders WN, Westerhoff HV, van Spanning RJ (2004). Expression of nitrite reductase in *Nitrosomonas europaea* involves NsrR, a novel nitrite-sensitive transcription repressor. Mol Microbiol.

[15] Hoglen J, Hollocher TC (1989). Purification and some characteristics of nitric oxide reductase-containing vesicles from *Paracoccus denitrificans*. J Biol Chem.

[16] Hooper AB (1968). A nitrite-reducing enzyme from *Nitrosomonas europaea*. Preliminary characterization with hydroxylamine ad electron donor. Biochim Biophys Acta.

[17] Spott O, Russow R, Stange CF (2011). Formation of hybrid N2O and hybrid N2 due to codenitrification: first review of a barely considered process of microbially mediated N-nitrosation. Soil Biol Biochem.

[18] Starkenburg SR, Chain PS, Sayavedra-Soto LA, Hauser L, Land ML, Larimer FW (2006). Genome sequence of the chemolithoautotrophic nitrite-oxidizing bacterium *Nitrobacter winogradskyi* Nb-255. Appl Environ Microbiol.

[19] Sutka RL, Ostrom NE, Ostrom PH, Breznak JA, Gandhi H, Pitt AJ (2006). Distinguishing nitrous oxide production from nitrification and denitrification on the basis of isotopomer abundances. Appl Environ Microbiol.

[20] Sutka RL, Adams GC, Ostrom NE, Ostrom PH (2008). Isotopologue fractionation during N_2_O production by fungal denitrification. Rapid Commun Mass Spectrom.

[21] Heil J, Wolf B, Brüggemann N, Emmenegger L, Tuzson B, Vereecken H (2014). Site-specific ^15^N isotopic signatures of abiotically produced N_2_O. Geochim Cosmochim Ac.

[22] Kozlowski JA, Price J, Stein LY (2014). Revision of N_2_O-producing pathways in the ammonia-oxidizing bacterium *Nitrosomonas europaea* ATCC 19718. Appl Environ Microbiol.

[23] Caranto JD, Lancaster KM (2017). Nitric oxide is an obligate bacterial nitrification intermediate produced by hydroxylamine oxidoreductase. Proc Natl Acad Sci U S A.

[24] Kozlowski JA, Kits KD, Stein LY (2016). Comparison of nitrogen oxide metabolism among diverse ammonia-oxidizing bacteria. Front Microbiol.

[25] Upadhyay AK, Hooper AB, Hendrich MP (2006). NO reductase activity of the tetraheme cytochrome C554 of *Nitrosomonas europaea*. J Am Chem Soc.

[26] Shoun H, Fushinobu S, Jiang L, Kim S-W, Wakagi T (2012). Fungal denitrification and nitric oxide reductase cytochrome P450nor. Philos Trans R Soc B.

[27] Kester RA, De Boer W, Laanbroek HJ (1997). Production of NO and N_2_O by pure cultures of nitrifying and denitrifying bacteria during changes in aeration. Appl Environ Microbiol.

[28] Dundee L, Hopkins DW (2001). Different sensitivities to oxygen of nitrous oxide production by *Nitrosomonas europaea* and *Nitrosolobus multiformis*. Soil Biol Biochem.

[29] Caranto JD, Vilbert AC, Lancaster KM (2016). *Nitrosomonas europaea* cytochrome P460 is a direct link between nitrification and nitrous oxide emission. Proc Natl Acad Sci U S A.

[30] Zhu-Barker Xia, Cavazos Amanda R., Ostrom Nathaniel E., Horwath William R., Glass Jennifer B. (2015). The importance of abiotic reactions for nitrous oxide production. Biogeochemistry.

