# Supplementary material for: Indications for enzymatic denitrification to N2O at low pH in an ammonia-oxidizing archaeon
Source: ISME J. 2019 Jun 21;13(10):2633–8. doi: 10.1038/s41396-019-0460-6 (PMC6775971; doi:10.1038/s41396-019-0460-6)
Supplement: Supplementary file 1 — supplementary information [file 41396_2019_460_MOESM1_ESM.docx]

**Supplementary Information**

**Indications for enzymatic denitrification to N_2_O at low pH in an ammonia-oxidizing archaeon**

Man-Young Jung^1,2^, Joo-Han Gwak^1^, Lena Rohe^3^, Anette Giesemann^4^, Jong-Geol Kim^1^, Reinhard Well^4^, Eugene L. Madsen^5†^, Craig W. Herbold^2,6^, Michael Wagner^2,6,7^ and Sung-Keun Rhee^1*^

Running title: N_2_O formation by AOA at low pH

^1^Department of Microbiology, Chungbuk National University, 1 Chungdae-ro, Seowon-Gu, Cheongju 362-763, South Korea

^2^University of Vienna, Center for Microbiology and Environmental Systems Science, Division of Microbial Ecology, Althanstrasse 14, A-1090 Vienna, Austria

^3^Helmholtz Centre for Environmental Research – UFZ, Department of Soil System Sciences, Theodor-Lieser-Strasse 4, D-06120 Halle (Saale), Germany

^4^Thünen Institute of Climate-Smart Agriculture, Bundesallee 50, D-38116 Braunschweig, Germany

^5^Department of Microbiology, Cornell University, Ithaca, New York 14853-8101, USA

^6^Comammox Research Platform of the University of Vienna

^7^Department of Biotechnology, Chemistry and Bioscience, Aalborg University, Denmark

^†^ Deceased Aug, 9^th^, 2017

^*^To whom correspondence should be addressed.

E-mail: rhees@chungbuk.ac.kr. Phone: 82-43-261-2300. Fax: 82-43-264-9600.

**This document includes:**

Supplementary Materials and methods

Supplementary Tables S1 to S4

Supplementary Figures S1 to S4

Supplementary References

**Supplementary Materials and Methods**

*Cultivation of ammonia-oxidizing microorganisms*

An artificial freshwater medium (AFM), whose composition and preparation are detailed elsewhere [1], was used for the cultivation of pure cultures of AOA (*Nitrosotenuis chungbukensis* MY2 [2], and *Nitrosocosmicus oleophilus* MY3 [1]) and AOB (*Nitrosomonas europaea* ATCC 19718). AFM was amended with 0.1 ml of trace element mixture, 0.1 ml of Fe-NaEDTA solution, and 0.1 ml of vitamin solution [1]. Sodium bicarbonate and HEPES were added to AFM to final concentrations of 2 mM and 3 mM, respectively. Sodium pyruvate (0.5 mM) was only added to the medium for strain MY2. All AOM were routinely grown chemolithoautotrophically in the respective medium containing 1 mM NH_4_Cl (but a lower substrate concentration was used for the experiments; see below).

For the N_2_O studies, cultures of AOA and AOB were incubated with 50 ml of AFM in 120 ml serum bottles sealed with a butyl rubber stopper (Bellco, USA). The initial cell concentrations of strain MY3, strain MY2 and strain 19718 were ca. 1.5 × 10^5^, 2.8 × 10^6^ and 1.8 × 10^6^ ml^-1^, respectively, in all experiments except for initial cell concentrations of strain MY3 at pH 5.5 in Supplementary Figure S2 (ca. 5 × 10^5^ ml^-1^). All of the ammonia-oxidizing microorganism (AOM) cultures were incubated in the dark at 30 °C with unmodified ambient air in the head-space. Sampling for gas and chemical analysis, and supplementation with chemicals was conducted using gas-tight syringes. The resistance of the butyl rubber stopper against leakage of N_2_O gas for 1 month was confirmed using standard gas. The cultures were supplemented with 0.2 mM NH_4_Cl as the sole energy source. For co-culture experiments, selected AOM were incubated together with the NOB, *Nitrobacter winogradskyi* Nb-255. This NOB was pre-grown in a recommended medium (www.atcc.org/mediapdfs/480.pdf) and then the cells were washed two times using AFM and re-suspended with the same volume of AFM. The cell suspension (5%, v/v) of NOB was inoculated into AFM together with the respective ammonia oxidizer strain. SYBR Gold staining was used for microscopic counts of total fluorescent cells after the cells were filtered (0.2 µm polycarbonate GTTP membranes; Merck Milipore, Germany) as described previously [1]. The ammonia concentration was determined photometrically [1]. Nitrite and nitrate concentrations were determined photometrically by the acidic Griess reaction [1, 3, 4]. The N_2_O yield after the completion of ammonia oxidation was determined based on the ammonia consumed and nitrite produced from the same cultures.

The specific growth rate was calculated by determining the slope according to the equation μ = (ln*N*_1_ – ln*N*_0_)/(t_1_ – t_0_), where (ln*N*_1_ – ln*N*_0_) is the change in the natural log of the ammonia concentration and (t_1_ – t_0_) is the change in time. At least four points in time were used for each growth rate calculation.

To determine the strain-specific ranges of pH used in N_2_O-production assays, AOM cultures were prepared in HEPES (pH 6.5-8.5) and HOMOPIPES buffer (pH 5.5-6.0) with pH adjusted using 1 M HCl or 1 M NaOH. For all strains and conditions, the pH did not significantly change (< 0.1) during the experiments with these buffered media. The lowest pH selected for each strain was the one found to support a growth rate that was 20% of the strain’s optimal growth rate. For all strains examined, N_2_O was analyzed after 0.2 mM ammonia was oxidized completely. The ammonia level was monitored by measuring ammonia in the AFM every 2 days. Dissolved O_2_ concentrations (µM) in the nitrifying media were continuously monitored with Oxygen Spot Sensors (OXSP5, Pyroscience GmbH, Aachen, Germany) glued to the inner wall of the vials. The sensors were operated with an optical oxygen meter (FireStingO2, Pyroscience GmbH, Aachen, Germany) linked to a computer with Pyro Oxygen Logger software.

*Isotopic signature analysis of N_2_O*

The headspaces of the 120 ml serum bottles were used for the isotopic N_2_O analysis and for calculating the N_2_O production yield. Gas samples were taken (14 mL) from the headspace and transferred into 12 mL exetainer vials (Labco Ltd, Lampeter, UK). In dependence on the N_2_O concentration up to four gas samples were taken from the headspace of one bottle. N_2_O concentration was analysed by gas chromatography (GC) with a helium ionization detector (HID) (Agilent 7890A, Santa Clara, USA).

To estimate whether the produced N_2_O derives mainly from nitrification (hydroxylamine) or nitrifier denitrification, N_2_O was analyzed by isotope ratio mass spectrometry (IRMS). The dual isotope and isotopomer signatures of N_2_O were determined as described before by Lewicka-Szczebak *et al.* [5], i.e., δ^18^O of N_2_O (δ^18^O-N_2_O), average δ^15^N (δ^15^N^bulk^-N_2_O), and δ^15^N from the central N-position (δ^15^N^α^-N_2_O), after cryo-focusing by IRMS using a Delta V IRMS (Thermo-Fisher, Bremen, Germany), which allowed simultaneous detection of *m/z* 44, 45, and 46 of the intact N_2_O^+^ as well as *m/z* 30 and 31 for NO^+^ fragment ions [6]. The IRMS was connected to a gas chromatograph (GC) (Trace GC Ultra, Thermo–Fisher, Bremen, Germany) and a modified Precon [7] (Thermo- Fisher) equipped with an autosampler (model Combi-PAL, CTC-Analytics, Zwingen, Switzerland) [6]. Relative ratios of the two ^15^N-substituted isotopomers (^15^R^α^ = ^14^N^15^NO/^14^N^14^NO and ^15^R^β^ = ^15^N^14^NO/^14^N^14^NO) were determined. This measurement enabled calculation of δ^15^N^β^: δ^15^N^β^ = 2 × δ^15^N^bulk^ − δ^15^N^α^, and ^15^N site preference of N_2_O produced (SP): SP = δ^15^N^α^ - δ^15^N^β^ [8]. The dual isotope and isotopomer ratios of a sample (R_sample_) were expressed as the ‰ deviation from ^15^N/^14^N and ^18^O/^16^O ratios of the reference standard materials (R_std_), atmospheric N_2_, and standard mean ocean water (SMOW), respectively:

δX = (R_sample_/R_std_ − 1) × 1000,

where X = ^15^N^bulk^, ^15^N^α^, ^15^N^β^, or ^18^O of N_2_O [9]. The typical analytical precision levels were 0.2, 0.4, and 0.3‰ for δ^15^N^bulk^, δ^15^N^α^, and δ^18^O of N_2_O produced, respectively.

The amount of N_2_O necessary for a proper analysis was collected from gas samples through freezing in liquid N_2._ Using exetainer vials (12 mL volume), the N_2_O concentration has to be higher than 3 ppm to supply enough N_2_O. Hence, due to very low N_2_O concentration in some samples, N_2_O was collected by freezing N_2_O from two, three or four parallel gas samples, respectively. For comparability reasons, standard N_2_O gas was measured in parallel to the samples after collection from one to four exetainer vials. Accuracy and precision were comparable to standards treated as usual. We therefore can exclude any negative impacts of freezing N_2_O from multiple samples on the quality of the IRMS analysis. The δ^15^N value of NH_4_^+^ was measured by combustion of the pure salts (ammonium chloride, Sigma-Aldrich) using an elemental analyzer coupled to an IRMS (DeltaPlus, Thermo-Finnigan, Bremen, Germany).

The abiotic control incubations were prepared with 50 ml of AFM with various concentrations of chemicals (see Supplementary Table S2) in 120 ml serum bottles sealed with the same butyl rubber stopper used in the biotic N_2_O experiments (see above). In addition, experiments were performed with active AOA inoculations (*N. oleophilus* MY3) in the presence of the inhibitor chlorite [10] and ammonia oxidation and cell growth were not observed a month after initiating the incubations (data not shown). The amount of N_2_O gas in the headspace of the abiotic control incubations (Table S2) was measured after one month of incubation using a GC with a Quadrupole Mass Spectrometer (QMS) (6890A (G1540A)/5973N, Agilent, USA). The QMS recorded the relative quantitative intensity of ions according to their mass-to-charge ratios (*m/e*). The N_2_O ion peaks produced by electron impact ionization were measured in the *m/e* positions and their background in the mass spectrometer was subtracted. Gas samples were taken from the headspace of culture bottles using a 5 ml gas-tight syringe and injected via a gas sampling valve using a 1 ml sample loop in the GC. The GC was fitted with a 4 m stainless steel column packed with Porapak Q (80/100 mesh, Restek, USA) to separate N_2_O and CO_2_ from the sample gas. The oven was isothermal at 50°C and the N_2_O ion peaks were measured three times for each sample. We used six certified reference gas mixtures of N_2_O in nitrogen, i.e., 0.198, 0.331, 0.550, 0.798, 12.0, and 94.9 N_2_O μmol/mol, to calibrate the GC/QMS. These reference gas mixtures were prepared gravimetrically by the Korea Research Institute for Standards and Science and verified by international comparisons, i.e., CCQM-K68 in 2010 [11].

*N_2_O tracer experiment*

The N_2_O tracer experiment was performed using the same culture conditions as for the isotope signature analysis. However, for the tracer experiments 0.2 mM of NH_4_Cl was used as the sole electron donor in the presence of 0.2 mM of 98 atm% Na^15^NO_2_ (Cambridge Isotope Laboratories), which was added at the start of the experiment to determine the ^15^N atom incorporation from nitrite into N_2_O. Experiments were established with pH values of 6.5-8.5 at intervals of 0.5 for the AOA strain *Nitrosotenuis chungbukensis* MY2 and AOB strain *N. europaea* 19718, and with pH values of 5.5-8.5 at intervals of 0.5 for *Nitrosocosmicus oleophilus* MY3. The amount of N_2_O gas in the headspace was measured after 0.2 mM ammonia was oxidized completely for all strains and conditions using the same method as for the abiotic control experiment. Given the presence of unlabeled NH_4_^+^ and ^15^N-labeled NO_2_^-^ in these experiments, there were three possible combinations of N isotopes for N_2_O: ^14,14^N_2_O (*m/e* 44), ^14,15^N_2_O (*m/e* 45), and ^15,15^N_2_O (*m/e* 46). The QMS recorded the relative quantitative intensity of ions according to their mass-to-charge ratios (*m/e*). The N_2_O ion peaks produced by electron impact ionization were measured in these three *m/e* positions and their background in the mass spectrometer was subtracted.

*Phylogenetics of cytochrome P450 genes*

All publicly available genomes were downloaded from Genbank on August 5^th^, 2017 and screened using hmmsearch [12] against the P450 Pfam (PF00067), using an expect value cutoff of 10^-3^. The sub-genic part of the gene that aligned to the pfam hmm was reciprocally screened against the Pfam-A database and only those that hit the PF00067 hmm as its best-hit model over at least 200 amino acids were retained for phylogenetics. This dataset was supplemented with all sequences from the CYPED database (https://cyped.biocatnet.de) and clustered using usearch [13] requiring 70% identity and a query coverage of at least 90%. All sequences from nitrifiers (*Thaumarchaeota*, proteobacterial AOB, *Nitrospira)* were re-added to the dataset, if they did not form a centroid in Usearch. Centroids and all nitrifier sequences (12.533 sequences in total) were aligned using mafft [14] and their phylogeny was reconstructed using IQTREE with the WAG model of amino acid substitution for 5000 iterations.

*Expression of the cytochrome P450 gene* in *N. oleophilus*

Total RNA was extracted from cells harvested from 500 ml of exponentially growing culture at pH 5.5 and 7.5 using the RNeasy Mini Kit (Qiagen, Germany) according to the manufacturer’s recommendations and residual DNA was removed from the eluents using the RNase-free DNase Set Kit (Qiagen) and the DNase-treated samples were purified using RNeasy MinElute Kit (Qiagen). RNA was eluted to 30 µL of RNase-free water. 10 µL of the eluent was used for subsequent reverse transcription procedures and the rest of the eluent was stored in -20 °C and later subjected to qPCR to check for DNA contamination. cDNA was synthesized using the SuperScript First Strand synthesis system (Invitrogen, San Diego, CA) with RNaseOUT solution (40 U μL^-1^; Invitrogen) according to manufacturer’s instructions. Concentrations of RNA and cDNA were determined using an ND-1000 spectrophotometer (NanoDrop Technologies, Wilmington, DE).

To generate qPCR standards for the two cytochrome P450 genes (MY3_00641 and MY3_01637) in *N. oleophilus* MY3, PCR primers were designed that match at starting and ending positions of the ORFs. A set of primers that hybridized within cytochrome P450 genes was designed to amplify cDNA (for primer sequences see Supplementary Table S4). Quantitative real-time PCR experiments were carried out using a MiniOpticon real-time PCR detection system (Bio-Rad Laboratories, Hercules, CA) and Opticon Monitor Software version 3.1 (Bio-Rad Laboratories, Hercules, CA).

Expression level of cytochrome P450 genes were compared with those of housekeeping genes encoding (i) two enzymes involved in CO_2_ fixation [methylmalonyl-CoA mutase large subunit (MY_02370) and 4-hydroxybutyryl-CoA dehydratase (MY3_03315)], (ii) 16S rRNA, and AmoA. The respective primer sets for qPCR standard generation and qPCR analyses of expression levels of the housekeeping genes are given in Supplementary Table S4. Thermal cycling parameters for amplification of cytochrome P450 genes were 15 min at 95 °C, followed by 40 cycles at 95°C for 20 s, 55°C for 20 s and 72°C for 20 s, with readings recorded after each cycle. PCR efficiency was 87-95% with r^2^ values ≥ 0.99 for all assays. In addition to housekeeping-gene-transcript-based normalization, total genomic DNA (i.e., total cell)-based normalizations were performed. Total genomic DNA was extracted from an aliquot of cells harvested from the same exponentially growing culture at pH 5.5 and 7.5 used for total mRNA extraction. Genes of AmoA and 16S rRNA were quantified from the total genomic DNA for estimation of total cells of the cultures used for preparation of the cDNA.

*Statistical analyses*

Statistical analyses were performed using the computing environment R (http://www.R-project.org/) and SigmaPlot 11.0 (Systat Software Inc., San Jose, CA, USA). Differences between SP values of N_2_O at various pH conditions for AOM culture were analyzed by one-way analysis of variance (ANOVA). The significance level (α) for ANOVAs was 0.05. Tukey's comparison of means then served to identify significant condition effects. In addition, the t-test was used to test for significance of the difference in expression of cytochrome P450 transcripts at pH 5.5 and 7.5.

**Supplementary Table S1**. The concentration of N_2_O produced from cultures of AOA and AOB. Measuring the concentration of N_2_O was performed after 0.2 mM ammonia was oxidized completely for all strains under each condition. Each result represents the mean of at least duplicate experiments.

| **Strain and pH condition** | **N_2_O (N_2_O-N; nmol)** |
| --- | --- |
| ***Strain MY2*** |  |
| pH 6.0 | 80.03 ± 2.82 |
| pH 7.5 | 18.25 ± 4.85 |
| ***Strain MY3*** |  |
| pH 5.5 | 40.07 ± 5.41 |
| pH 6.5 | 29.16 ± 1.64 |
| pH 7.5 | 19.15 ± 1.63 |
| pH 8.5 | 15.73 ± 0.85 |
| ***Strain MY3 + NOB*** |  |
| pH 5.5 | 28.51 ± 1.31 |
| pH 7.5 | 18.50 ± 0.75 |
| ***Strain 19718*** |  |
| pH 6.5 | 103.97 ± 8.52 |
| pH 7.5 | 36.79 ± 2.75 |
| pH 8.5 | 32.69 ± 3.15 |
| ***Strain 19718 + NOB*** |  |
| pH 6.5 | 58.07 ± 3.15 |
| pH 8.5 | 31.32 ± 4.95 |

**Supplementary Table S2.** N_2_O production by abiotic control incubations. All control incubations were performed in the same growth medium (AFM) that was used for the biotic experiments, but without AOA inoculations. The indicated compounds were added to the AFM medium. In addition, experiments were performed after inoculation with *N. oleophilus* MY3, in the presence of the inhibitor chlorite [10]. N_2_O concentration measurements were performed a month after initiating the incubations. Each result represents the mean of at least duplicate experiments.

| **Media composition** | **N_2_O (N_2_O-N; nmol)** | |
| --- | --- | --- |
|  | **pH 5.5** | **pH7.5** |
| AFM* | 2.68 ± 0.12 | 2.67 ± 0.11 |
| 500 μM NH_4_^+^ | 2.65 ± 0.13 | 2.54 ± 0.02 |
| 500 μM NO_2_^-^ | 2.71 ± 0.22 | 2.70 ± 0.16 |
| 500 μM NO_3_ | 2.69 ± 0.18 | 2.65 ± 0.13 |
| 10 μM NH_2_OH | 2.68 ± 0.17 | 2.74 ± 0.49 |
| 50 μM NH_2_OH | 3.90 ± 0.31 | 7.85 ± 0.92 |
| 500 μM NH_2_OH | 10.2 ± 1.17 | 11.29 ± 2.22 |
| 500 μM NH_4_ + 500 μM NO_2_^-^ | 2.70 ± 0.22 | 2.71 ± 0.13 |
| 10 μM NH_2_OH + 500 μM NO_2_^-^ | 2.60 ± 0.19 | 2.39 ± 0.48 |
| 50 μM NH_2_OH + 500 μM NO_2_^-^ | 4.06 ± 0.40 | 10.49 ± 0.26 |
| 500 μM NH_2_OH + 500 μM NO_2_^-^ | 90.95 ± 7.69 | 185.14 ± 10.38 |
| 500 μM NH_4_ + 100 μM ClO_2_^-^ + 10% active strain MY3 inoculum | 2.71 ± 0.23 | 2.70 ± 0.33 |
| 10 μM NH_2_OH + 500 μM NO_2_ + 100 μM ClO_2_^-^ + 10% active strain MY3 inoculum | 2.68 ± 0.12 | 3.47 ± 0.60 |
| 50 μM NH_2_OH + 500 μM NO_2_ + 100 μM ClO_2_^-^ + 10% active strain MY3 inoculum | 6.57 ± 0.14 | 14.60 ± 1.70 |
| 500 μM NH_2_OH + 500 μM NO_2_ + 100 μM ClO_2_^-^ + 10% active strain MY3 inoculum | 129. 64 ± 10.04 | 210.92 ± 11.05 |

* AFM, artificial freshwater medium as a control for the abiotic treatments

Supplementary Table S3. Isotopic characteristics of N_2_O produced by ammonia-oxidizing microorganisms at various pH conditions. All experiments used air-equilibrated media with an assumed δ^18^O-O_2_ of ca. 23.5‰ versus Vienna Standard Mean Ocean Water (VSMOW). The δ^15^N values are reported as ‰ versus air, while the δ^18^O values are reported as ‰ versus VSMOW. The isotope values for N_2_O are the isotopic composition of the N_2_O produced after correcting for background material. δ^18^O-H_2_O was –8.95 (1.10) ‰. δ^15^N-NH_4_^+^ was –1.20 (0.10) ‰.

| **Strain** | **pH** | **Replicate** | **δ^18^O vs SMOW** | **δ^15^N_bulk_ vs air (‰)** | **δ^15^N_α_ vs air (‰)** | **δ^15^N_β_ vs air (‰)** | **SP (‰)** |
| --- | --- | --- | --- | --- | --- | --- | --- |
| MY2 | 6.0 | 1 | 32.27 | –13.15 | 0.78 | –27.04 | 27.82 |
|  |  | 2 | 31.70 | –13.71 | –0.27 | –27.10 | 26.83 |
|  |  | 3 | 31.72 | –13.99 | –0.08 | –27.87 | 27.79 |
|  |  | 4 | 31.72 | –13.80 | 0.41 | –27.96 | 28.37 |
|  | 7.5 | 1 | 33.35 | –11.47 | 3.31 | –26.20 | 29.51 |
|  |  | 2 | 33.49 | –11.90 | 2.37 | –26.13 | 28.49 |
|  |  | 3 | 32.92 | –11.77 | 2.92 | –26.41 | 29.33 |
|  |  | 4 | 33.39 | –11.64 | 2.41 | –25.63 | 28.04 |
| MY3 | 5.5 | 1 | 36.32 | –14.07 | 0.42 | –28.52 | 28.94 |
|  |  | 2 | 36.78 | –13.79 | 0.33 | –27.87 | 28.20 |
|  |  | 3 | 36.62 | –13.58 | 0.72 | –27.84 | 28.55 |
|  |  | 4 | 35.94 | –14.43 | 0.65 | –29.45 | 30.10 |
|  | 6.5 | 1 | 38.11 | –11.83 | 0.96 | –24.58 | 25.54 |
|  |  | 2 | 37.98 | –12.95 | 0.69 | –26.55 | 27.25 |
|  |  | 3 | 37.83 | –12.06 | 0.89 | –24.96 | 25.85 |
|  |  | 4 | 37.59 | –9.92 | 3.90 | –23.69 | 27.59 |
|  | 7.5 | 1 | 36.76 | –0.77 | 13.26 | –14.76 | 28.02 |
|  |  | 2 | 36.99 | –2.11 | 12.21 | –16.40 | 26.47 |
|  |  | 3 | 36.49 | 1.07 | 12.21 | –10.05 | 26.62 |
|  |  | 4 | 36.30 | 1.11 | 11.87 | –9.63 | 24.84 |
|  | 8.5 | 1 | 37.48 | 2.69 | 6.98 | –1.59 | 25.52 |
|  |  | 2 | 38.18 | –11.65 | 14.04 | –13.44 | 24.94 |
|  |  | 3 | 38.54 | –12.78 | 9.66 | –15.30 | 22.86 |
| MY3 + NOB | 5.5 | 1 | 38.02 | –3.48 | 11.49 | –18.41 | 29.91 |
|  |  | 2 | 37.81 | –4.32 | 10.69 | –19.28 | 29.97 |
|  |  | 3 | 38.05 | –4.55 | 10.38 | –19.44 | 29.83 |
|  |  | 4 | 37.78 | –2.30 | 11.57 | –16.13 | 27.70 |
|  | 7.5 | 1 | 37.86 | –2.32 | 9.65 | –14.25 | 23.90 |
|  |  | 2 | 37.65 | 0.85 | 13.96 | –12.21 | 26.17 |
|  |  | 3 | 37.12 | 0.90 | 13.99 | –12.15 | 26.14 |
|  |  | 4 | 37.48 | 2.50 | 13.98 | –8.93 | 22.91 |
| ATCC 19718 | 6.5 | 1 | 17.95 | –41.23 | –41.97 | –40.49 | –1.49 |
|  |  | 2 | 17.96 | –41.17 | –41.73 | –40.60 | –1.13 |
|  |  | 3 | 18.77 | –39.68 | –38.54 | –40.82 | 2.28 |
|  |  | 4 | 18.75 | –40.10 | –39.01 | –41.19 | 2.19 |
|  | 7.5 | 1 | 28.93 | –5.96 | 8.62 | –20.49 | 29.11 |
|  |  | 2 | 29.00 | –5.78 | 7.73 | –19.26 | 26.99 |
|  |  | 3 | 29.22 | –5.40 | 8.31 | –19.07 | 27.38 |
|  |  | 4 | 29.50 | –5.33 | 8.79 | –19.42 | 28.21 |
|  | 8.5 | 1 | 26.18 | –4.74 | 10.46 | –19.90 | 30.36 |
|  |  | 2 | 26.30 | –8.28 | 6.52 | –23.03 | 29.55 |
|  |  | 3 | 27.30 | –5.24 | 9.20 | –19.63 | 28.83 |
|  |  | 4 | 26.14 | –5.92 | 9.68 | –21.47 | 31.15 |

**Supplementary Table S4.** Sequences of oligonucleotide primers used for qPCR analysis of cytochrome P450 transcription in *N. oleophilus* MY3

| **Primer**  **(Position and target ORF of primer extension)** | **Target gene** | **Sequence (5’ to 3’)** | **Application** |
| --- | --- | --- | --- |
| Full-00641-F | Cytochrome P450 | ATGTCAAAGACAATACTAGA | qPCR standard |
| Full-00641-R | Cytochrome P450 | TCATATCGATCTAATCCTTG | qPCR standard |
| Full-01637-F | Cytochrome P450 | ATGAAAGTGAAATCAAATAT | qPCR standard |
| Full-00641-R | Cytochrome P450 | TCAATCATTAAATCTCTTCA | qPCR standard |
| 40-00641-F | Cytochrome P450 | GAATTTCCTCCAGGACCA | qPCR |
| 40-01637-F | Cytochrome P450 | GTGTATCCACCAGGACCGCGC | qPCR |
| 420-00641-R | Cytochrome P450 | CCATGATGCGGCCATCTTCCC | qPCR |
| 420-01637-R | Cytochrome P450 | CTACAATTGTCCCATCTCTCC | qPCR |
| 946-00641-F | Cytochrome P450 | GCAGGACACGAAACCACATC | qPCR |
| 946-01637-F | Cytochrome P450 | GCAGGGCATGAGACGACTGC | qPCR |
| 1246-00641-R | Cytochrome P450 | TGCATAACATATTGACTCAT | qPCR |
| 1246-01637-R | Cytochrome P450 | TGCATTAGATACTGACTCAT | qPCR |
| Full-02370-F | Methylmalonyl-CoA mutase large subunit | ATGACGCTGCCCAAATTTG | qPCR standard |
| Full-02370-R | Methylmalonyl-CoA mutase large subunit | TCATGGTGGTAGCTCGAGAC | qPCR standard |
| Full-03315-F | 4-hydroxybutyryl-CoA dehydratase | ATGCCTATTAAAAATGGGTC | qPCR standard |
| Full-03315-R | 4-hydroxybutyryl-CoA dehydratase | CTATTTTTTTATTCCAAATATCC | qPCR standard |
| 320-02370-F | Methylmalonyl-CoA mutase large subunit | CGCGTTCGACCTCGCTACCCA | qPCR |
| 540-02370-R | Methylmalonyl-CoA mutase large subunit | GTACCGGATAACTTCTCCGG | qPCR |
| 683-03315-F | 4-hydroxybutyryl-CoA dehydratase | CTATGGAAGACAATCGTGCGA | qPCR |
| 892-03315-R | 4-hydroxybutyryl-CoA dehydratase | GATCAATACATCTCCTAATCCGG | qPCR |

**Supplementary References**

1. **Jung MY, Kim JG, Sinninghe Damste JS, Rijpstra WI, Madsen EL et al.** A hydrophobic ammonia-oxidizing archaeon of the *Nitrosocosmicus* clade isolated from coal tar-contaminated sediment. *Environ Microbiol Rep* 2016;8(6):983-992.

2. **Kim JG, Park SJ, Sinninghe Damste JS, Schouten S, Rijpstra WI et al.** Hydrogen peroxide detoxification is a key mechanism for growth of ammonia-oxidizing archaea. *Proc Natl Acad Sci U S A* 2016;113(28):7888-7893.

3. **Miranda KM, Espey MG, Wink DA**. A rapid, simple spectrophotometric method for simultaneous detection of nitrate and nitrite. *Nitric Oxide* 2001;5(1):62-71.

4. **Griess-Romijn van Eck E**. Physiological and chemical tests for drinking water. *NEN* 1966;1056 IV-2.

5. **Lewicka-Szczebak D, Augustin J, Giesemann A, Well R**. Quantifying N_2_O reduction to N_2_ based on N_2_O isotopocules – validation with independent methods (helium incubation and ^15^N gas flux method). *Biogeosciences* 2017;14(3):711-732.

6. **Well R, Flessa H**. Isotope fractionation factors of N_2_O diffusion. *Rapid Commun Mass Spectrom* 2008;22(17):2621-2628.

7. **Brand WA**. PreCon: A fully automated interface for the pre-GCconcentration of trace gases on air for isotopic analysis. *Isot Environ Healt S* 1995;31(3-4):277-284.

8. **Yoshida N, Toyoda S**. Constraining the atmospheric N_2_O budget from intramolecular site preference in N_2_O isotopomers. *Nature* 2000;405(6784):330-334.

9. **Coplen TB**. Guidelines and recommended terms for expression of stable-isotope-ratio and gas-ratio measurement results. *Rapid Commun Mass Spectrom* 2011;25(17):2538-2560.

10. **Jung MY, Well R, Min D, Giesemann A, Park SJ et al.** Isotopic signatures of N_2_O produced by ammonia-oxidizing archaea from soils. *ISME J* 2014;8(5):1115-1125.

11. **Lee JB, Lee JS, Moon DM, Kim JS, Wessel R et al.** Final report on international comparison CCQM-K68: nitrous oxide in synthetic air. *Metrologia* 2011;48(1A):08004.

12. **Eddy SR**. Accelerated Profile HMM Searches. *PLoS Comput Biol* 2011;7(10):e1002195.

13. **Edgar RC**. Search and clustering orders of magnitude faster than BLAST. *Bioinformatics* 2010;26(19):2460-2461.

14. **Katoh K, Standley DM**. MAFFT multiple sequence alignment software version 7: improvements in performance and usability. *Mol Biol Evol* 2013;30(4):772-780.

**Supplementary Figure S1.** N_2_O yields at various pH conditions for (A) the AOB *N. europaea* 19718, (B) the AOA *N. chungbukensis* MY2 and (C) the AOA *N. oleophilus* MY3. The concentrations of N_2_O produced and ammonia oxidized were measured at the start of the experiment and after 0.2 mM ammonia was completely oxidized. The concentration of dissolved O_2_ at the start and end of the experiments was at ~230 µM and > ~218 µM, respectively. Co-culture experiments with *Nitrobacter winogradskyi* Nb-255 were also performed for strain MY3 and strain 19718. In all experiments, ammonia was stoichiometrically converted to nitrite or nitrate (co-cultures). Each data point represents the mean of at least triplicate experiments. In some cases, the error bars are smaller than the symbols. The raw data used in this plot are presented in Supplementary Table S1.

**Supplementary Figure S2.** Stoichiometric conversion of ammonia to nitrate or nitrite, respectively, by a co-culture of *N. oleophilus* MY3 and the NOB *N. winogradskyi* Nb-255 (A and B), and by *N. oleophilus* MY3 alone (C and D) at pH 7.5 (A and C) and 5.5 (B and D). Each data point represents the mean of triplicate experiments. Initial pH of each culture was adjusted to 7.5 and 5.5. The initial cell concentrations of strain MY3 for the pH 7.5 and 5.5 were ca. 1.5 × 10^5^ and ca. 5 × 10^5^ ml^-1^, respectively.

**Supplementary Figure S3.** Comparative phylogenetic analysis of cytochrome P450 genes of strain MY3 and other nitrifiers. The dataset of nitrifier P450 genes was supplemented with the CYPED database ([cyped.biocatnet.de](http://cyped.biocatnet.de/)), which was clustered using usearch [13] requiring 70% identity and a query coverage of at least 90%. The phylogeny was reconstructed using IQTREE with the WAG model of amino acid substitution for 5000 iterations. Locations of non-*Nitrosocosmicus* P450 genes from nitrifiers are indicated by letters (A-M). Bars are sized according the number of (published) nitrifier genomes with a cytochrome P450 branching at the lettered position and colored according to taxonomic affiliation indicated in the legend. Bars are further labeled with the number of genomes represented. The attached table summarizes the P450 gene incidence in nitrifier genera.


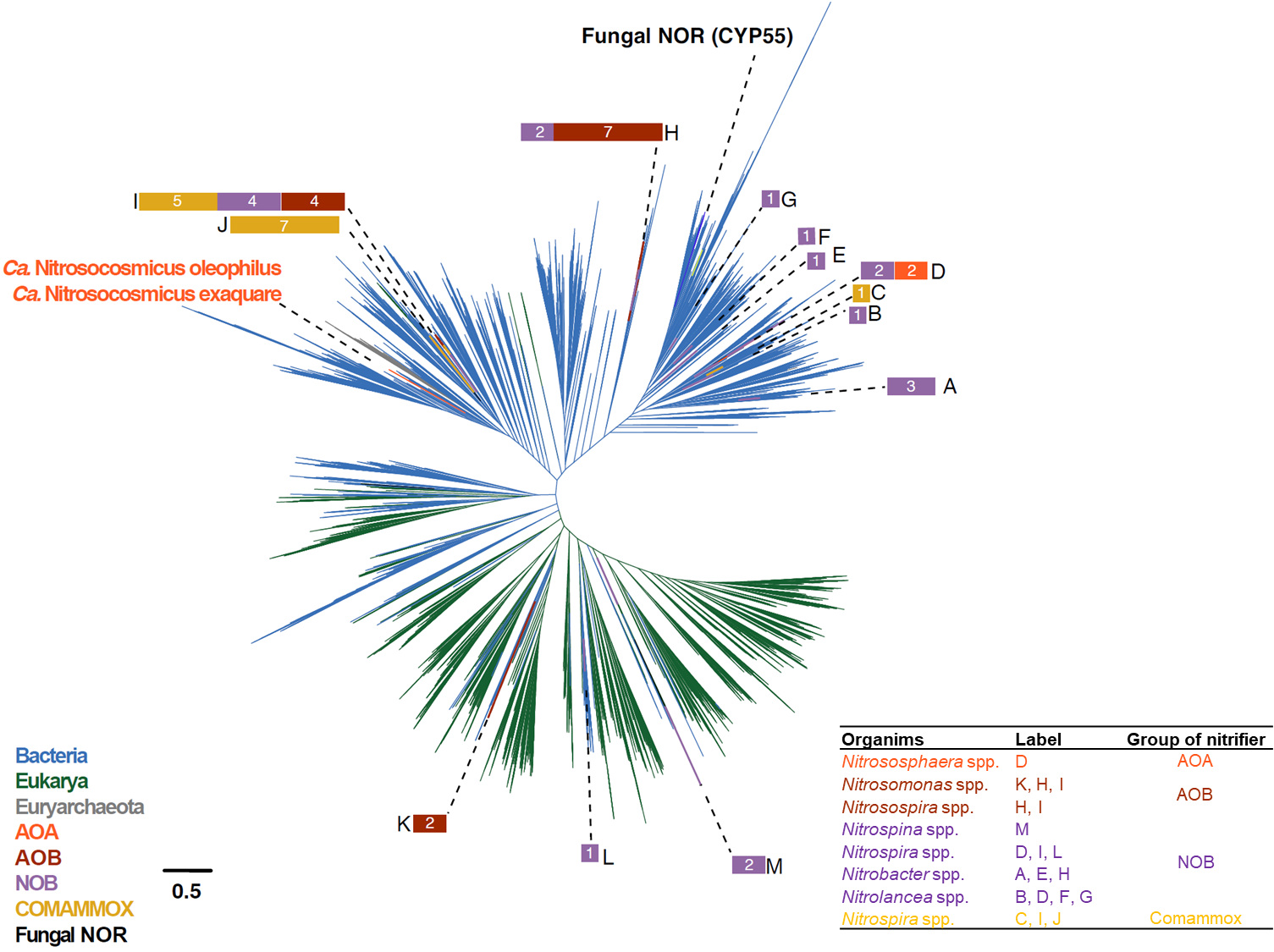


**Supplementary Fig. S4.** *N. oleophilus* MY3 cytochrome P450 expression ratios at two different pH conditions (pH 5.5 and pH 7.5) normalized to the number of cells. Mean values of two independent qPCR experiments performed on reverse-transcribed total RNA from cells grown at pH 5.5 and 7.5 and harvested at exponential stage are presented. Transcript abundance of 16S rRNA, *amoA*, two CO_2_ fixation genes [methylmalonyl-CoA mutase large subunit (MY_02370), 4-hydroxybutyryl-CoA dehydratase (MY3_03315)], and two different copies of cytochrome P450 gene (locus: MY3_00641 and MY3_01637) were normalized to 16S rRNA gene copies of total genomic DNA of the cultures used for cDNA preparation. Due to the loss of RNA during RNA extraction all values are small. The ratio of relative genes expression at pH 5.5 to 7.5 is indicated in red. Error bars represent the standard errors of triplicate incubations. Significance of differences between pH 5.5 and 7.5 for each gene was determined by the t-test (*: *P* < 0.5, **: *P* < 0.1 and ***: *P* < 0.05).
